# Engaging with Families Is a Challenge: Beliefs among Healthcare Professionals in Forensic Psychiatric Care

**DOI:** 10.1155/2015/843717

**Published:** 2015-09-10

**Authors:** Ulrica Hörberg, Eva Benzein, Christen Erlingsson, Susanne Syrén

**Affiliations:** ^1^Department of Health and Caring Sciences, Faculty of Health and Life Sciences, Linnaeus University, 351 95 Växjö, Sweden; ^2^Department of Health and Caring Sciences, Faculty of Health and Life Sciences, Centre for Collaborative Palliative Care, Linnaeus University, 351 95 Växjö, Sweden

## Abstract

Being healthcare professionals in the complex field of forensic psychiatry care (FPC) seems particularly challenging. Historically, families have almost been invisible in FPC. The aim of this study was to uncover beliefs among healthcare professionals concerning families of patients admitted for FPC. Using a hermeneutical approach inspired by Gadamer's philosophy, group interviews with healthcare professionals in four Swedish forensic psychiatric clinics were analyzed. Analysis resulted in seven key beliefs. There were three beliefs about families: family belongingness is a resource for the patient; most families are broken and not possible to trust; and most families get in the way of the patient's care. Four beliefs concerned encounters with families: it is important to achieve a balance and control over the family; it is essential to set aside one's own values and morals; family-oriented work is an impossible mission; and family oriented work requires welcoming the families. Despite ethical dilemmas of working with families in FPC, healthcare professionals showed a willingness and desire to work in a more family-oriented manner. More knowledge, understanding, and caring tools are needed in order to meet the needs of the family as well as support the family's resources.

## 1. Introduction

Forensic psychiatry is a complex field of care, due to the dual nature of carrying out of caring actions in an involuntary care situation. The patients have committed crimes under the influence of serious mental disorder and are often cared for over long periods of time in an institutional environment with a high level of security [1]. An individual care plan has to be drawn up for every patient according to the Swedish Forensic Psychiatric Care Act [2]. Furthermore, the Swedish National Board of Health and Welfare [3] highlights the importance of relationships and interactions between patients in forensic psychiatric care and their families. Guidelines emphasize the importance of providing family members of patients with opportunities for participating in care planning for their relative. This thus indicates that family members are considered important in increasing the patient's chances of living a well-functioning life after discharge from the psychiatric facility. Since there is no legal requirement concerning the involvement of the patient's family in the care planning, their participation is thus greatly dependent on the attitudes and willingness to invite families to be involved in caring of the healthcare professionals. Historically, families have had an almost invisible role in the forensic psychiatric care (FPC) context [4]. In general, the specific situation of the families in relation to FPC is not adequately taken into consideration in existing FPC care and rehabilitation programs [5]. When viewed from a family nursing perspective, this complex care field should also involve the patients' family members whenever possible. One study involving a national sample of Swedish nurses showed that they generally have a positive view of involving family members in patients' care [6]. But does this outlook also hold true for nurses and other staff who work in forensic psychiatry care?

## 2. Background

### 2.1. Forensic Psychiatric Care

Forensic psychiatry is in the interface between mental health and the law and can be understood as assessment and treatment of people with mental disorders who show antisocial or violent behavior [7]. There are a number of different security levels in forensic psychiatric clinics, such as maximum, high, medium, and low levels [8, 9]. In Sweden, FPC is the care context for patients with serious mental disorders, who have committed crimes and have been referred for treatment in accordance with the Forensic Psychiatric Care Act, instead of being sentenced to prison [2]. The overall goal for the forensic psychiatric care in Sweden is to prevent new crimes and avoid acts of violence by the patients [10] and to consider the safety of the country's citizens. Further goals are to improve the patients' health processes and to enable, if possible, the patient's return to a “normal” life in the community.

FPC staff have to simultaneously manage caring for patients while maintaining control [1, 11–18]. These studies highlight the complexity in forensic psychiatric caring related to the dilemma of providing custodial care, that is, providing care in a custodial manner. Due to the contradiction of simultaneously providing care whilst guarding and containing the patients, the role of a healthcare professional in a FPC context appears to be particularly challenging [11, 12, 19]. While registered nurses and licensed assistant mental health nurses provide for the patients' daily care needs on the ward, other healthcare professionals, such as physicians, psychologists, occupational therapists, and social workers, have responsibility for specific treatment efforts. All these professionals have to some degree to interact with the patients' families.

### 2.2. Forensic Psychiatric Care and Families

Against the background of the complexity of FPC, it has been shown that when healthcare professionals involve families in FPC it has positive effects, not only for the patient but also for their family members and relationships within the family [20]. Research in the field of forensic psychiatry describes a multifaceted and extremely complex situation with an extensive impact on families. These families experience difficulty in maintaining family relationships, which is compounded by enforced separation between patients and their families, the geographical distance between the institution and the family home [4, 21], the security routines, and the organizational and cultural characteristics of the forensic psychiatric care units [22].

Matters are further complicated in that family members are themselves often victims of crimes committed by the patient [4, 21]. MacInnes [23] highlights that family members of patients in FPC experience a greater degree of burden than family members of patients in other psychiatric care contexts. Levels of violence reported by caregivers (either as victims of patient violence or witnessing the patient perform acts of violence) are higher and more dangerous for family members of FPC patients than for family members of nonforensic patients. In one study of a rehabilitation program it was stated that family members' needs included information about their relatives' symptoms of mental illness and their impact on everyday life [24]. It has been shown that healthcare professionals in FPC have less contact with family members than healthcare professionals in other psychiatric care contexts have with family members. Consequently, family members of patients in forensic psychiatric care experience that they are neither included in decisions about their relative's future nor provided with information. MacInnes [23] also found that family members' experiences and coping responses concerning their relative's situation and FPC are directly affected by the beliefs expressed by healthcare professionals in FPC about family role and the patient's illness and treatment.

In spite of having knowledge about strained family situations, nurses in FPC have been found to have little collaboration with patients' family members [25]. A review of the literature reveals a paucity of research focusing on the family and healthcare professionals' beliefs when one family member is cared for in FPC. Further no research at all could be located specifically concerning healthcare professional's attitudes and beliefs about families when engaging with families in FPC. The three studies that were located have been conducted in a FPC context among healthcare professionals with a focus on the significance of attitudes and/or beliefs for FPC. One study by Pulsford and coworkers [26] explored attitudes and beliefs towards patient aggression in a high secure unit among staff and patients. Staff and patients had opposing views about how to understand and to handle aggression. The staffs' beliefs were also found to be of importance for whether they managed situations by using interpersonal approaches or by controlling strategies. Forsyth et al. [27] found that healthcare staff on a forensic psychiatric rehabilitation unit were influenced by their beliefs when giving advice to patients about nutrition. In a study on beliefs and attitudes among staff towards patients in medium and low secure forensic mental health settings, no significant differences between security levels were found. Differences were, however, found where males reported more negative attitudes in relation to blame and avoidance, and younger participants demonstrated more negative attitudes than older participants in relation to fear and danger [28].

Though few in number, these studies indicate that attitudes and beliefs among healthcare professionals are important elements influencing their choices of approaches and actions in care. It is conceivable that attitudes and beliefs also influence why healthcare professionals do not include families in FPC.

### 2.3. Beliefs

A beliefs framework as described below provides the basis for developing knowledge in this study. A belief can be understood as a figure of what is “truth”, what actually is, and what would be ideal. Beliefs can thus make a significant contribution to understanding phenomena as well as impacting and understanding behavior [29]. Beliefs can also be understood as social representations and thus as being developed collectively. When a person expresses his/her beliefs about a phenomenon into words and when another person gives these words similar meanings, these persons can then be said to share a common belief about that phenomenon. Such shared beliefs become knowledge that allows individuals to explain, understand, and evaluate phenomena in a similar way [30]. Against this theoretical background, it is reasonable to assume that beliefs exist and develop collectively among healthcare professionals within the FPC context and have a major impact on whether and how families are recognized and included in patients' care.

## 3. Aim

The aim of this study was to uncover beliefs among healthcare professionals concerning families that have contact with forensic psychiatric care.

## 4. Method

The study has a hermeneutical approach inspired by the philosophy of Gadamer [31] and based on his maxim that a hermeneutic attitude requires researchers to maintain optimal openness and flexibility. The methodological procedure consists of a constant movement between the texts as a whole and its parts in order to gain a new whole, with new understandings and meanings [32]. Gadamer [31] assumed that it is only through one's preunderstandings that a deepened understanding is possible. He argued that one has to be aware of the preunderstanding of a phenomenon and at the same time strive to suspend the preunderstanding in order to be open-minded and discover anything new, that is, the otherness of something. This implies that researchers need to be aware of their personal preunderstandings. Both data collection and analysis were thus characterized by an open questioning and critical attitude. Furthermore, a self-critical stance with a vigilant awareness of current preunderstandings was maintained in order to prevent a constraining influence that might affect the findings.

### 4.1. Settings and Participants

The study settings were four Swedish forensic psychiatric clinics: one maximum security clinic, one high security clinic, and two clinics with medium security level. The patients admitted to the clinics were referred for treatment in accordance with the Forensic Psychiatric Care Act [2].

An information letter was distributed to staff with the help of key persons from each clinic (such as head nurses). A convenience sample was formed from healthcare professionals who were interested in participating in the study and stated their interest in a written response letter to the researchers. A total of 9 men and 15 women, between 25 and 65 years old (mean age 46 yrs) participated in the study. Their work experience as caregivers in FPC varied from 1.5 to 38 yrs (mean 18.5 yrs). The participants included one occupational therapist, 1 social worker, 5 registered nurses (RNs), 11 licensed assistant mental health nurses, and 6 social pedagogues. The latter worked together with nurses on the ward and had responsibility for social pedagogical activities.

### 4.2. Data Collection

Data was collected in six group interviews (three at the maximum security unit, one at the high security unit, and two at the medium security units). The number of participants in the group interviews ranged from two to seven. Interviews were conducted in rooms separate from the FPC unit and focused on participants' experiences about family relationships and staff-family-patient interactions in a FPC context. The interviews were characterized by an inviting attitude where the participants were provided with the opportunity to share their experiences. Open questions were asked in order to capture the participants' experiences and to gain rich descriptions of family issues [33]. Interviews started with the following question: which significance do the relationships between patients and their family members have for forensic psychiatric care? Further open-ended questions were then asked in order to elicit examples from each informant about their experiences of working with families such as can you describe a situation where you have encountered a patient's family? When needed, additional follow-up questions were asked, for example, “can you tell me more about…?,” “what happens in the situation?,” or “what does that mean to you?” With an approach to obtain variations and nuances, participants were also invited to reflect on each other's responses and compare own experiences with those described by others in the group.

Of the four researchers involved in data collection, one researcher was present at all interviews and the other three were cointerviewers in varying constellations. During the interviews, one of the researchers had the main responsibility for conducting the interview. A second researcher assisted in making sure that the interview remained focused on the interview topic; in keeping track so that all of the participants were given opportunities to express what and how much they wanted; and by asking additional relevant questions not previously addressed in the interview. Interviews each lasted approximately one and a half hours, were recorded, and were transcribed verbatim.

### 4.3. Analysis

Data analysis was an interpretative procedure inspired by Gadamer [31] and was characterized by a continuously movement between the whole and the parts like a hermeneutical spiral [32]. The question “what beliefs stood out from the interview in participants' statements?” guided the analysis of beliefs concerning families in contact with forensic psychiatric care. In order to capture the “beliefs” more specifically questions were asked of the text such as what is the underlying meaning in the participants' statements, what appear as subjective “truths,” and how reasonable do the interpretations appear to be in a FPC context?

A text comprised of the transcribed interviews was read several times in order to gain an understanding of the text as a first whole and to begin to discern how parts of the text reflected and deepened understanding of the whole. Literal statements were in particular identified and preliminarily noted as clearly defined or partly hidden beliefs. These preliminary noted beliefs were compared in order to distinguish their similarities and differences, and several areas of beliefs concerning family in contact with FPC were then identified.

### 4.4. Ethical Considerations

Written permission to conduct the study was provided by the directors of each forensic psychiatric clinic. Both written and oral informed consent was obtained from all participants who had also received a letter with information about the study before the interview took place. Participation was voluntary and participants were informed about their right to decline further participation without having to provide any motivation or risking any reprisals. Principles of anonymity, integrity, and confidentiality have been maintained and the study conforms to the Declaration of Helsinki [34]. The study was approved by the Ethics Committee at Linköping University, Sweden (reg. number 2011/228-21).

## 5. Findings

The analysis uncovered two main areas of beliefs held by healthcare professionals in FPC, namely, beliefs about* families* and beliefs about* encounters with families*. These two main areas included seven beliefs that were interpreted in the whole of the data, across all interviews, and understood as shared beliefs among the healthcare professionals in FPC. Thus it also seems reasonable to understand them as collectively developed [26]. These beliefs are conceptualized as* key beliefs* unlocking professionals' understanding of family issues connected to their work in FPC and as essential in professional identity and for understanding the FPC context.

Three key beliefs are about* families*: family belongingness is a resource for the patient; most families are broken and not possible to trust; and most families get in the way of the patient's care. Four key beliefs concern* encounters with families*: it is important to achieve a balance and control over the family; it is essential to set aside one's own values and morals; family-oriented work is an impossible mission; and family-oriented work requires welcoming the families.

### 5.1. Beliefs about Families

#### 5.1.1. Key Belief: Family Belongingness Is a Resource for the Patient

A family provides a sense of belonging and security and is essential if patients are to experience meaning and hope in their lives. Families in contact with FPC have a dream about the ideal family. It is thus extremely unfair that FPC can entail that patients grow older without contact with their family or lack knowledge of how the family is managing in the world outside the clinic. To be part of a family, preferably a large family, is beneficial for the development of the patients' care. Good family relationships also provide energy and stimulate a longing to recover and be released from FPC.

Belonging to a family is pivotal for patients' recovery and reintroduction in society, as well as for patients existential well-being. Forensic psychiatric patients, however, often have few family relationships and for those patients who have someone left it is usually a parent. The lack of contact with other family members during time outside the unit and upon release from care entails a danger for patients. Existential problems in connection with being discharged from care are greater when the patient is not part of a family anticipating his/her return.

They have greater existential problems in… well, in just sharing their concerns… there are so many…. “what can I do here?” and “what's going to happen?” Maybe they have nowhere to live and might not have any city or town they call home. Things that are so basic for anyone else. They are sort of “displaced.” And I think that it's rather obvious that patients who have someone somewhere don't have those kinds of worries. It might not be a close relation but at least there is someone.

#### 5.1.2. Key Belief: Most Families Are Broken and Not Possible to Trust

There is often a complex interplay within families who have contact with FPC. This, however, depends on the illness and criminal history in families as well as problems connected to maintaining family relationships in a high security context. It is necessary, as a healthcare professional, to be able to relate to patients and family members from widely varied family constellations and with complex and difficult family situations. Moreover, it is often impossible to trust family members to assist in achieving the objectives in the patient's care plan. Regardless of how families seem to be, it is still important to accept them as they are.

…in my experience… if one has something to say against the family in front of the patient… it is almost like fighting against God… it doesn't work… You must have the viewpoint that the family is like it is… just as you have your own family…. And saying that it is either this way or that way doesn't work… that's the whole thing… otherwise you never make any progress with anything else… and for that matter, you don't get to know anything either… everybody just clams up… nobody wants to reveal anything.

#### 5.1.3. Key Beliefs: Most Families Get in the Way of the Patient's Care

Families in contact with FPC seem to fit in categories such as ordinary, dangerous, uncooperative and incapable. It is, however, difficult to establish favorable contacts with patients' family members. The nature of the family is pivotal for how or even if contact with them can be established.

The type of family preferred by healthcare professionals in forensic psychiatric care are traditional families, persons who belong together through blood and legal ties, most often including parents, siblings, and children. If patients want to include other persons in their care and care planning sessions that do not match this description, matters become complicated and made more difficult.

Then there were other persons that patients themselves wanted to be involved. But it is difficult to know where to draw the line. If it is friends it feels like one doesn't want to…well… how much should I tell? Obviously, this is something they can decide themselves…. Who is participating in a care planning session for example? But it's really been without any boundaries too. That friend can be my support person. It's complicated. Now this sounds really negative and there are some really good examples of supportive significant others… but I don't think it's all that often.

The ordinary families were found among the traditional families, the former are the ones that have just had bad luck in life. As staff you can identify with the ordinary family and can see how easy it could have been for oneself to have ended up in the same situation as such a family. “I myself am a parent. //So it occurred to me that it could have been me sitting there, it could have been me!”

Family members, in ordinary families, are committed and willing to participate in care and to help the patient as much as they can. When rules, routines, work methods, and guidelines are correctly followed by staff, these families were not difficult and were thus allowed a more participatory role in FPC.

Most of the families, however, diverge from what can be considered ordinary, such as families where threatening and violent situations occur, either connected to the patient's illness or connected to earlier events. These families are viewed as dangerous families and the staff found it difficult to figure out how to relate to these families.

But we shouldn't assume that role either… of being like a defense attorney for patients …as if she (patient) is also a crime victim… We as staff can think, “Oh, she is so nice and sweet now”… but, like …it's not our place to get involved in that stuff… it really isn't.

There are also uncooperative families with troublesome and demanding family members, as well as those who are “… manipulative and just know which buttons to press… and who always are one step ahead in the game.” Patients from such families pay less attention to staff and more attention to family members who have negative opinions and who question care and treatment. These are families who retract into themselves, exclude staff involvement, and take things into their own hands, actions that impede the successful carrying out of the patient's care and treatment. Patients in a suitable care environment should not be aware of or involved in situations where family members and staff have different opinions about what and how care is provided. There is a need to get these family members to take sides with the staff.

The incapable families can be distinguished from other families by their problems in comprehending the necessity and relevance of the care provided. It is difficult and takes a long time for patients in FPC to understand why treatment is needed and the intentions and purposes of the care. To understand and work through the situation is even harder for family members. Some families seem incapable of understanding the patient's problems and have difficulties in getting a grip on them or seeking more information. Sometimes there is even a general lack of interest in being involved in decision making related to the patient's care.

### 5.2. Beliefs about Encounters with Families

#### 5.2.1. Key Belief: It Is Important to Achieve a Balance and Control over the Family

Usually it is positive that families get together and have the opportunity to work through their conflicts. As a healthcare professional, you should assist families with their relational problems. However, sometimes the violence and threatening atmosphere can be so serious that it is necessary to limit the possibilities to meet for the family members and the patients.

When working with families you evaluate how the patient and the family members are influencing each other, that is to say, if they are good or bad for each other. In addition, working with families means assessing the patient's needs and considering whether to allow or to limit the family's and patient's influence on each other. In order to make progress in the patient's care it is essential to facilitate understanding between patient and family members and support families' engagement and involvement in the patient's care.

At one care planning session there was* [sic]* a father and a mother and the son. The father was really focused on the son getting a job because then everything would be OK. We tried to explain that the son needs to get in to supported housing; that he can't manage on his own out in society and the son himself thinks that the idea of a supported housing sounds great, one can tell. In the end the father came around and could also see that. That was sort of special because it felt like the dad was so much against us initially in the meeting. He changed his mind, which was nice…

#### 5.2.2. Key Belief: It Is Essential to Set Aside One's Own Values and Morals

Positive meetings within and with families are made more difficult by the work environment on forensic psychiatry units. It is not only family relationships and internal functioning that make it complicated to work in FPC. Laws and regulations always have priority. Laws governing care and treatment are not adapted to the reality of the care situation for the patient or for family members. Care is provided under coercion, and patients are sentenced for crimes. This entails the constant pressure of thinking about security. This also has an effect on and makes it harder to work with families. Supporting family relationships must thus take a back seat to the pursuance of laws and regulations. Routines, for example, surveillance protocols, are given priority in forensic psychiatry and cause suffering in the family as well as being experienced as offensive.

It's so mortifying! I can imagine how it feels for a family member, as a child, to come to see your father who you haven't seen for ages and having to come here. Just having to go through the security gates, back and forth, and then going up [to the visitor's room]. No toys, nothing. And sitting there together with staff… there in the same room to watch and all the rigmorale* [sic]*…

When more control and surveillance than necessary is being used it feels uncomfortable; like being in the wrong place. “You feel like… Goodness, it's awful. I wish I didn't have to even be here… they would have done so much better on their own…” It's always emotionally draining being the one who has to supervise a visit. Staff presence can have a negative effect on the family's opportunity to act naturally.

… but we still have to do our job, at the same time we are only human, so we empathize with all this, and still need to be there and be seen and be as unobtrusive as possible and still be alert.

An adjustment to the forensic psychiatry laws and regulations also entails not having authorization to handle situations in a flexible way. Providing care in accordance with specified prerequisites implies the necessity to put one's own and family's needs and feelings to the side. Not being able to influence care situations is an obstacle. On the other hand, the fact that “we [staff] do not own the problem,” lifts away the heavy responsibility of not being able to provide the care we consider appropriate and makes it possible to cope with experiencing tragic and difficult family situations.

#### 5.2.3. Key Belief: Family-Oriented Work Is an Impossible Mission

Although a clear work description, authorization, or any kind of complete mandate for working with families does not exist, there are expectations that a family-oriented perspective should be in place even though no appreciation of such work is apparent. These contradictory messages in the forensic psychiatry unit lead to uncertainty about one's own capability to work with families.

… One doesn't have the self-confidence to do the job like one really can, and one isn't fully allowed to do it either. There is a hierarchy in the system that doesn't really allow that knowledge to be put to use. It permeates our self-confidence, how we work…

The will to improve working from a family-oriented perspective is there, but both the knowledge and the resources are lacking.

We can't just go in and do something we don't know what we are doing…//… because that is a damned important job… one should begin with respect that is what is needed…. In order to do the job, and not just keep it on the level of superficial cordiality.

At the same time planned interactions with families are not very common in forensic psychiatric care due to a lack of interest in and/or not seeing the importance of working with families. “Those of us employed here who want to work with families are probably not such a large group.” Lack of clarity concerning what the task is and uncertainty concerning authorization and capabilities, together with the laws regulating security in forensic psychiatry, are a hindrance for providing care. All contribute to passivity in working with families. “You do what is required, but nothing more…” At the same time working with families is an impossible mission; the staff maintain that they have to exert themselves in the encounters with the patients' families in order to provide the best prerequisites for the patient's recovery.

#### 5.2.4. Key Belief: Family-Oriented Work Requires Welcoming the Families

It is important to implement new routines and new ways of interacting in FPC in order to be successful in working with families. The legally required care planning session is an example of a routine that should be changed. There are too many persons involved in them. Smaller, more secure, and inviting contexts would facilitate trusting relationships between staff and family members. There is also a need for improved access and continuity in contact with families.

If only we had been able to have that on the docket, to involve families in an ongoing and more structured way. “You are welcome if you have time”. It must be arranged and booked. Otherwise it doesn't happen. For example it might coincide with family members' job schedules.

The general mindset on forensic psychiatry units and individual attitudes must change concerning families, family member's role, and the opinions about involving family members in the care of their relative.

… This is where I think we are deficient, although it was worse before. I mean that we just didn't see families as a resource or source of knowledge even though they were close to the patient. … it isn't just meetings, it's also about how we must have a genuine interest to learn more from those in the patient's network. Otherwise, we tend to hold these meetings because we are supposed to, that we hold them “for forms sake”. Sometimes one gets the feeling that we aren't giving it our all.

## 6. Discussion

The analysis uncovered two main areas of beliefs held among healthcare professionals in FPC, namely, beliefs about* families* and beliefs about* encounters with families*.

The key belief,* family belongingness is a resource for the patient*, appears to be a benchmark for what is important for the patients and as essential in their possibilities for experiences of meaning and hope. Another key belief was that* most families in contact with FPC are broken families not possible to trust*. These families were labeled as dangerous, uncooperative or incapable and described, for example, as difficult, troublesome, manipulative, or uninterested in getting involved in FPC. Believing that family belongingness is a resource for the patient is thus often inconsistent with the reality of the forensic psychiatry context, especially when encountering complicated and even destructive relationships in the patients' families. The family as a resource for the patient's recovery was shown in a study where patients in forensic psychiatry who are being parents are discharged earlier than those who are not [35]. When a family member suffers from severe mental illness the family can facilitate recovery through providing moral and practical support, and motivation to recover. Family psychoeducation is, however, of importance when the family also can impede recovery through acting as a stressor, displaying stigma and lack of understanding [36].

The findings in this study demonstrate that healthcare professionals view their own “strengths” as necessary (e.g., professional knowledge, experience, and FPC context). The key belief that* you have to be able to achieve a balance and have control over the family* shows how those who work in FPC assume that their interpretations take precedence over the opinions or decisions of the families. They act as experts, convinced that they know what is best for the family and believe they are providing vicarious strength to the family. Patients' experiences of FPC include being exposed to staff's exertion of authority, power, and punishment [1]. Patients and staff can find themselves in a “game of power” between the carers and those who are cared for [37]. It is probable that power structures in the correctional framework of FPC have similar meanings for both patients and their families. Foucault [38] describes power as “… everywhere; not because it embraces everything, but because it comes from everywhere” (p. 93). “Where there is power, there is resistance…//…these play the role of adversary, target, support or handle in power relations” (p. 95). If power games infiltrate relationships, between staff and patients, or between staff and families, it will affect the outcome of FPC for patients. FPC then becomes a struggle against an ever-present resistance that does not favor patients, families, or staff. The staff's pigeonholing of families as uncooperative and incapable and the key belief that they have to achieve a balance and have control over the family are very likely consequences of such a power game and are related to issues of distrust arising from interactions with these families. Once again against the background of Foucault [38], it is very likely that families categorized by staff as uncooperative and incapable are particularly vulnerable in relation to the staffs' “power” and decisions. In a forensic psychiatry context, helping these families to strengthen their resources and thus alleviate their vulnerability appears to be of specific importance.

These findings substantiate the description of FPC as limited and often inhibited by the organizational and judicial framework of correctional care. Being governed by a constant awareness of security risks in encounters between patient and their family members leaves staff with a sense of being in the wrong place and experiencing discomfort. The dilemma of wanting families to have as natural a meeting as possible, but being required to follow security regulations, is a probable explanation for the key belief about the inevitability of setting aside one's own values and morals while working in FPC. This also exemplifies staff's contradictory commitments of both correcting behavior and caring for patients in FPC [37]. Gildberg and coworkers [14] described this dilemma in FPC as “trust and relationship-enabling” or “behavior- and perception-corrective.” Several studies exemplify the central dilemma of providing custodial correctional care and at the same time care for the patients by creating caring relationships [1, 11–13, 15, 17, 19, 39, 40]. In a highly controlled and security-oriented context such as forensic psychiatry, it is a manifest risk that an existential view of being human, regardless if it involves patients, families, or staff, is overshadowed and thus becomes invisible. The central dilemma in this study can be understood against the background of a discourse of contradictory beliefs about families and family involvement.

The key belief that* it is essential to set aside one's own values and morals* can be understood as an ethical dilemma that healthcare professionals in a forensic psychiatry have to face. Austin et al. [41] describe forensic psychiatry as a unique practice area with its complex ethical demands and like a “moral minefield.” It is difficult to act ethically in forensic psychiatry because of the competing obligations that staff have and their role as a “double agent.” Thus “a relational ethics approach to healthcare practice focuses on being* with*, as well as being * for*, patients, families, and other professionals” (p. 843) [41] could be an important guiding principle in FPC. Jacob and Foth [42] argue for the need for ethical discussion and emphasize that staff need to develop an ethical sensitivity and reflect on how the forensic psychiatric environment of care affects patients and families as well as themselves. Being true to oneself as well as to the patients and family members is fundamental in care from a caring science perspective. Dahlberg et al. [43] discuss Heidegger's ideas and argue that we are not separate from our concrete engagements with the world or with others. Separation implies limitations and vulnerabilities in relation to our existence in a particular time, place, and culture and in relation to language. They argue for the need of an existential view of what being human means in today's healthcare. We highlight an existential perspective in this study. This perspective is essential for an understanding of family situations as well as for an understanding of staffs' situations and perspectives, both when required to maintain surveillance and control during meetings between patients and their family members and when working for improved access and continuity in contact with families.

One of the key beliefs uncovered in this study is staff's belief that* family related work is an impossible mission*. This belief may be related to an uncertainty among the healthcare professionals, both regarding their mandate and capabilities to work with families, including their belief that what they have to offer is not enough. However, this belief can also be understood from a “power” perspective. Holmes [11] posits nurses as “subjects of power.” Yet, because nursing practice is circumscribed both by formal and informal regulations in the forensic psychiatric context, nurses are at the same time “objects of power.” Nursing practice in forensic psychiatry involves both punishing and providing care. Nurses act as “agents of the law” through a wide range of power techniques, for example, sovereign techniques that involve coercion [11]. One important question to ask is if the family can be encountered in any other way in a caring culture founded on correction and discipline. Jacob and Foth [42] contend that prevailing caring structures in forensic settings are characterized by sovereign power techniques. These authors argue for the need of ethical and political discussions in relation to forensic care. Such discussions entail being able to understand the complexities of this area of care, an understanding that would support changes that promote patients, families, and staffs' well-being.

What these findings reveal is that staff in FPC would be well served by developing a sensitivity and awareness of collective beliefs about engaging with families. A customized, educational family nursing program, suitable for the forensic psychiatry context, could assist healthcare professionals in FPC to challenge and even change their beliefs. Evidence that such an educational approach can be helpful for nurses is found in results from a study among nurses in a nonforensic context. After having participated in an education and training program in family nursing, participating nurses had changed their attitudes to families in care and also perceived meeting with families as less burdensome [44].

## 7. Methodological Considerations

Data were collected through six group interviews at four Swedish forensic psychiatric clinics with different security levels. Although the study was performed with a convenience sampling procedure a variation in participants' age, gender, and years of experience within a FPC context was achieved. This variation in the sample can be considered as a strength of the study in relation to the transferability and validity of the results. However, when volunteering healthcare professionals were included in the study it cannot be ruled out that those who are not interested in family issues were underrepresented in the study. The fact that two of the group interviews involved only two-three participants can be seen as a limitation of the study. Although the study was designed to include more participants in both of these groups, at the time the interview was to take place some presumptive participants were not allowed to leave the ward due to current security reasons. Having fewer participants than planned in these groups does not necessarily have to mean that interview quality was negatively affected. In these smaller groups each participant had more time and space to express their experiences of working with families.

When beliefs were understood as expressed in the dialogue between individuals [29], group interviews appear to be a fruitful data collection method. Group interviews are considered to contribute to stimulating participants to tell their own experiences when they listen to others' experiences [33]. The participants' individual and collectively held beliefs regarding families in FPC from the experiences are understood to be embedded [30]. Using group interviews in this study contributed to variation as well as nuances in central issues in the data. Furthermore, the interviewer, by picking up discussion threads and asking the participants questions, encouraged them to further discuss and compare their views and experiences of family relations and about working with families. During the interviews it appeared that participants were free to express what they wanted. In spite of this, it cannot be completely excluded that the group dynamics also affected individual participants to keep up with or adapt their statements to the predominant beliefs in the groups, regarding families in FPC.

The starting point for the analysis was the concept of beliefs. The findings thus need to be understood in this conceptual frame. The intention of this study was not to present an absolute truth but instead what appeared to be true and ideal among subjects. In accordance with Gadamer [31], our approach during the analysis was to understand the text in its specific context and to be as open-minded as possible. Against the background of what is emphasized as validity in hermeneutic research [32], the study's plausibility and meaningfulness can be seen as strengthened by the preliminary interpretations during the analysis process having been evaluated and challenged by other researchers with various contextual preunderstanding. Furthermore, the two main beliefs areas and the seven key beliefs appear to have an internal logic, incorporating meanings of the subjective understanding about families and family work in FPC. The contradictory beliefs were understood as a logical consequence of the contradictory FPC context of providing care as well as guarding and containing the patients.

At the same time it is necessary to bear in mind that no interpretation is final from a hermeneutic point of view. An interpretation can close the circle of understanding, but only for now [45]. Findings in this study should instead be considered to contribute to the hermeneutic spiral of increased understanding of engaging with families in FPC.

## 8. Conclusion

In conclusion, forensic psychiatric care is a complex field of care, carried out in the interface between mental health and the law. This care context affects staff's ability to work family-oriented in the care of patients. There is a willingness and desire among healthcare professionals to work in a more family-oriented manner, but also negative beliefs that hinder engaging with families. Professionals in FPC need greater knowledge, understanding, and caring tools, in order to meet the needs of the family as well as to support the family's resources. The challenge is to implement a family-oriented approach in forensic psychiatric care that can lead to strategies that have the potential to both strengthen the families' well-being and health processes and to contribute to staff experiencing encounters with families in contact with FPC as less emotionally draining.
